# Designer Exosomes for Targeted Delivery of a Novel Therapeutic Cargo to Enhance Sorafenib-Mediated Ferroptosis in Hepatocellular Carcinoma

**DOI:** 10.3389/fonc.2022.898156

**Published:** 2022-06-24

**Authors:** Xiaoju Li, Qianqian Yu, Runze Zhao, Xinyan Guo, Chenlin Liu, Kuo Zhang, Wangqian Zhang, Jinghan Liu, Jinzheng Yu, Shuning Wang, Qiang Hao, Weina Li, Wei Zhang, Meng Li, Yingqi Zhang, Cun Zhang, Yuan Gao

**Affiliations:** ^1^ State Key Laboratory of Cancer Biology, Biotechnology Center, School of Pharmacy, The Fourth Military Medical University, Xi’an, China; ^2^ Department of Oncology, Tongji Hospital, Tongji Medical College, Huazhong University of Science and Technology, Wuhan, China

**Keywords:** hepatocellular carcinoma, resistance, sorafenib, engineered exosome, ferroptosis

## Abstract

Sorafenib is one of the few effective first-line drugs approved for the treatment of advanced hepatocellular carcinoma (HCC). However, the development of drug resistance is common among individuals with HCC. Recent evidence indicated that the anticancer activity of sorafenib mainly relies on the induction of ferroptosis. Furthermore, in our study, genes that suppress ferroptosis, especially GPX4 and DHODH, were enriched in sorafenib-resistant cells and primary tissues and were associated with poor prognosis of HCC patients who received sorafenib treatment. Therefore, a new ferroptosis inducer comprising a multiplex small interfering RNA (multi-siRNA) capable of simultaneously silencing GPX4 and DHODH was created. Then, exosomes with high multi-siRNA loading and HCC-specific targeting were established by fusing the SP94 peptide and the N-terminal RNA recognition motif (RRM) of U1-A with the exosomal membrane protein Lamp2b. The results from the *in vitro* and *in vivo* experiments indicate that this tumor-targeting nano-delivery system (Exo^SP94-lamp2b-RRM^-multi-siRNA) could enhance sorafenib-induced ferroptosis and overcome sorafenib resistance. Taken together, HCC-targeted exosomes (Exo^SP94-Lamp2b-RRM^) could specifically deliver multi-siRNA to HCC tissues, enhance sorafenib-induced ferroptosis by silencing GPX4 and DHODH expression and consequently increase HCC sensitivity to sorafenib, which opens a new avenue for clinically overcoming sorafenib resistance from the perspective of ferroptosis.

## Introduction

Hepatocellular carcinoma (HCC) was ranked as the sixth most commonly diagnosed cancer and the fourth leading cause of cancer-related death worldwide in 2018 (1, 2). In the early stages of HCC, curative treatment can be achieved with tumor ablation, resection, or liver transplantation (3). However, the majority of HCC patients are already in the middle or late stages at the time of diagnosis, missing the optimal window for curative treatment. Sorafenib is the first systemic therapy shown to improve survival in HCC and has been approved by the U.S. Food and Drug Administration (FDA) for treatment of unresectable HCC (4). Despite their initial response, sorafenib-treated tumors rarely regress completely, and most patients develop disease progression. Therefore, to improve the survival and quality of life of patients with HCC, combination therapies should be considered as a potentially superior treatment option.

Ferroptosis is a recently discovered form of programmed cell death characterized by iron-dependent accumulation of lipid peroxides to lethal amounts (5). A growing amount of evidence indicates that ferroptosis can be induced by inhibiting cystine/glutamate transporter (system x_c_-) activity, downregulating GPX4 or DHODH expression, and accumulating reactive oxygen species (ROS) (6). Recent reports have shown that sorafenib can induce ferroptosis by inhibiting system x_c_- (7). Moreover, numerous studies suggest that the anticancer activity of sorafenib mainly relies on inducing ferroptosis (5, 8–11). Therefore, targeting constituents of ferroptosis might be a promising strategy to increase sorafenib efficacy and overcome sorafenib resistance.

Herein, we first found that ferroptosis suppressor genes, especially GPX4 and DHODH, are enriched in sorafenib-resistant cells and primary tissues from patients and are associated with poor prognosis of HCC patients who receive sorafenib treatment. Then, we created a novel ferroptosis inducer comprising a multiplex small interfering RNA (multi-siRNA) suitable for simultaneously silencing GPX4 and DHODH. Then, exosomes (Exo^SP94-lamp2b-RRM^) with high tumor targeting ability and high multi-siRNA loading efficacy were constructed to deliver the multi-siRNA cargo. Using *in vitro* and *in vivo* models, we demonstrated that this tumor-targeting nanodrug (Exo^SP94-lamp2b-RRM^-multi-siRNA) could enhance sorafenib-induced ferroptosis and overcome sorafenib resistance, suggesting that it is a promising therapeutic strategy for treating sorafenib-resistant HCC.

## Materials and Methods

### Antibodies and Inhibitors

The antibodies used targeted the following proteins (dilutions used are included):

GAPDH (CW0101: immunoblotting, 1:1000) from CWBIOTECH; F-actin (40734ES75: immunofluorescence, 1:100) from YEASEN; U1A (10212: immunoblotting, 1:500) from Proteintech; CD63 (67605-1-Ig: immunoblotting, 1:5000) from Proteintech; CD9 (60232-1-Ig: immunoblotting, 1:5000) from Proteintech; TSG101 (14497-1-AP: immunoblotting, 1:1000) from Proteintech; GPX4 (67763-1-Ig: immunoblotting, 1:5000; IHC, 1:1000) from Proteintech; and DHODH (14877-1-AP: immunoblotting, 1:2000; IHC, 1:100) from Proteintech.

The inhibitors used are as follows:

Sorafenib (HY-10201: 10 μM for the *in vitro* assay and 30 mg/kg for the *in vivo* assay) and Ferrostatin-1 (HY-100579: 60 nM for the *in vitro* assay), and ferrostatin-1 (HY-100579: 5 mg/kg, intraperitoneal injection for the *in vivo* assay) were obtained from MedChem Express.

### Cell Lines and Culture

The HepG-2 and HEK-293T cell lines were obtained from the Type Culture Collection of the Chinese Academy of Sciences (Shanghai, China). These cell lines were authenticated by the analysis of short tandem repeat (STR) profiles, and all of them matched those of the standard cell lines in the DSMZ data bank. These cells tested negative for cross-contamination of other human cells and mycoplasma contamination. The sorafenib-resistant HepG-2 cell line (HepG-2R) was generated by continuous treatment of HepG2 cells with sorafenib up to 10 μM and maintained as previously described (12). HepG-2, HepG-2R and HEK-293T cells were cultured in DMEM containing 10% fetal bovine serum and 1% penicillin-streptomycin.

### Plasmid Construction

Briefly, the sequences for SP94 peptide (SFSIIHTPILPL), the CDS sequences of Lamp2b and the CDS sequences of the N-terminal RNA recognition motif (RRM) of U1-A were orderly arranged from N-terminal to C-terminal. Then the sequence encoding the above fusion protein was digested with BamHI-XhoI and cloned into pcDNA3.1(+) vector. The clones were confirmed by DNA sequencing and stored for the following experiments.

### Exosome Purification

For exosome isolation from HEK-293T cells, cells were transfected with control or SP94-Lamp2b-RRM expressing plasmids with Lipofectamine3000. 12 h later, cells were further cultured in DMEM with 10% exosome-depleted FBS (ultracentrifugation at 120,000 ×g for 16 h) for 24–36 h. Then, the culture medium was precleared by centrifugation at 500×g for 15 min and then at 10000×g for 20 min. Exosomes were isolated by ultracentrifugation at 110000×g for 70 min at 4°C and washed in PBS using the same centrifugation conditions. The concentration of exosomes was determined by measuring total exosomal protein using a Pierce BCA Protein Assay Kit (Thermo Scientific, USA) according to the manufacturer’s instructions. NanoSight and transmission electron microscopy were used to determine the size distribution, concentration and morphology of the exosomes. For siRNA loading experiments, 100 nM corresponding siRNAs were transfected with Lipofectamine 3000. Twenty-four hours later, the cells were further cultured in DMEM with 10% exosome-depleted FBS for another 24 h, followed by exosome isolation.

### Transmission Electron Microscope Assay

Cells were collected and fixed with 2.5% glutaraldehyde. Subsequently, cells were postfixed in 2% tetroxide and dehydrated through a series of gradient ethanol solutions. Samples were embedded in epoxy resin, cut into thin slices, and placed onto a nickel grid. Images were acquired using a Tecnai G2 Spirit transmission electron microscope (Thermo Fisher).

### Intracellular ROS Measurements

A lipid ROS assay was performed as described previously. Briefly, cells were incubated with PBS containing 10 µM DCFDA dye in a cell culture incubator for 30 min. Cells were then collected and washed twice with PBS followed by resuspension in 200 µl of PBS. ROS levels were analyzed using a Beckman CytoFLEX system through the FITC channel.

### Detection of Malondialdehyde (MDA)

Analysis of lipid peroxidation was assessed by quantifying the MDA concentration in cell lysates using a Lipid Peroxidation MDA Assay Kit (S0131, Beyotime) in accordance with the manufacturer’s instructions.

### Animal Study

Six-week-old, male, athymic BALB/c nude mice were used. All animal experiments were carried out under protocols approved by the Animal Care and Use Committee of Fourth Military Medical University.

For *in vivo* tracking of exosomes, purified exosomes with the indicated modifications were labeled with the fluorescent dye DiR at a final concentration of 8 μM (Invitrogen). Labeled exosomes were collected by ultracentrifugation after washing with saline and stored in saline before use. Mice were injected with labeled exosomes (100 μg at the protein level in 100 μL) *via* tail vein injection. Mice were subjected to fluorescent living imaging 6 h after injection with an *in vivo* imaging system (IVIS lumina II).

For orthotopic implantation, six-week-old male nude mice were anesthetized with 3% (w/v) pentobarbital sodium by intraperitoneal injection. Then, 2×10^6^ sorafenib-resistant HepG-2 cells stably expressing luciferase were surgically implanted into the left liver lobes of mice. Tumor growth was monitored by bioluminescence with an *in vivo* imaging system (IVIS lumina II). Two weeks after inoculation, mice were randomized to each group and began to receive different treatments. In the sorafenib treatment group, sorafenib (30 mg/kg) was given every 3 days. In the sorafenib and exosome combination treatment group, sorafenib was administered at the same dose, and 100 μg of the indicated exosomes (at the protein level) was injected *via* the tail vein 24 h after every sorafenib administration. At the end of the experiment, mice were sacrificed. Tumor bearing liver tissues and the main organs were collected for the following experiments.

### Immunohistochemistry

This experiment was performed as previously described (13). Briefly, sections (4 μm thick) of paraffin-embedded samples were deparaffinized and rehydrated in a graded series of ethanol. After inactivation of endogenous peroxidase activity with 3% H_2_O_2_ in methanol for 10 min, the sections were washed three times in PBS and blocked with goat serum for 20 min. Then, they were incubated with primary antibodies in a humid container at 4°C overnight. After the addition of the PowerVision™ complex, tissue sections were incubated at 37°C for 20 min followed by treatment with DAB. PBS in place of primary antibody was used as a negative control.

### Statistical Analysis

The data are presented as the mean ± s.e.m. from at least three independent experiments. Statistical analysis was performed using GraphPad Prism software. A random number table was used to randomize the mice into control and treatment groups. The numbers of mice were determined on the basis of our pretests and previous experience with similar experiments. A value of *P*<0.05 was considered statistically significant. The statistical tests were two-sided.

## Results

### Suppressed Ferroptotic Activity During Sorafenib Treatment Is Associated With Compromised Therapeutic Efficiency

**Figure 1:**
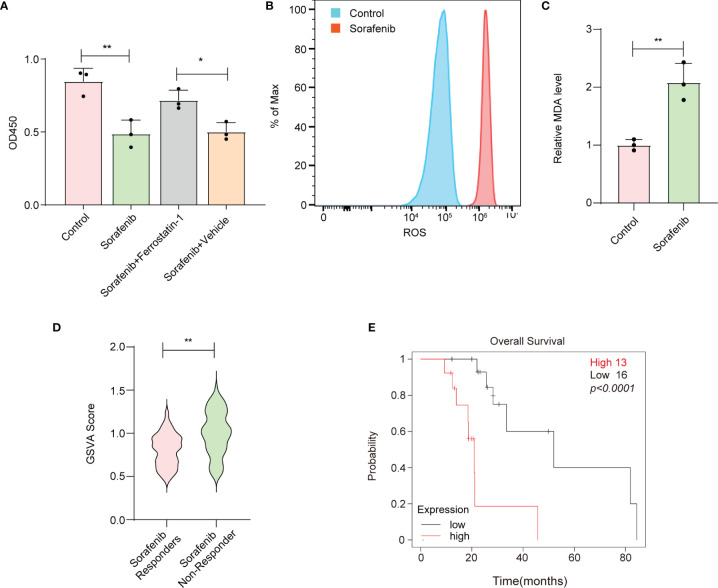
Reduced ferroptotic activity during sorafenib treatment is associated with compromised therapeutic efficiency. **(A)** Viability of HepG-2 cells as measured by the CCK-8 assay. Ferrostatin-1 is a ferroptosis inhibitor. Sorafenib (10 μM). **(B)** HepG-2 cells were treated with 10 μM sorafenib, and ROS production was assessed by DCFH-DA staining followed by flow cytometry. **(C)** MDA levels in HepG-2 cells treated with vehicle or 10 μM sorafenib for 24 h. **(D)** GSVA was conducted to calculate the score for enrichment of ferroptosis suppressor genes. HCC tissues from sorafenib responders or sorafenib nonresponders were obtained from the Gene Expression Omnibus (GSE1109211). **(E)** Kaplan–Meier analysis of overall survival based on the expression signature of ferroptosis suppressor genes. **(A, C)** The data are shown as the means ± S.E.M. **(A)** ANOVA with Dunnett’s t-test. **(C, D)** Unpaired t-test. **(E)** Log-rank test. *p < 0.05, **p < 0.01.

To confirm whether sorafenib could induce ferroptosis in HCC cells, we performed a CCK-8 assay. Our data showed that sorafenib-mediated cell death in the human HCC cell line HepG-2 was blocked by ferrostatin-1 (a ferroptosis inhibitor) (**Figure 1A**, **Figure S1A**). The accumulation of reactive oxygen species (ROS) and lipid peroxidation are key events in ferroptosis (14); thus, we analyzed the levels of ROS and the end products of lipid peroxidation (i.e., MDA) in HepG-2 cells. The results indicated that sorafenib increased the levels of ROS and MDA in HepG-2 cells (**Figures 1B, C**). Apart from ferroptosis-suppressing agents, numerous genes have been identified as key ferroptosis suppressors. For example, glutathione peroxidase 4 (*GPX4*) resides in the center of a network that functions to prevent the accumulation of lipid hydroperoxides, thereby strongly suppressing ferroptosis (15). Interestingly, we constructed a ferroptosis suppressor gene signature and discovered that ferroptosis suppressor genes were enriched in sorafenib-resistant cells and primary tissues from HCC patients (**Figure 1D**, **Figure S1B**). Moreover, the results indicated that the high expression level of the ferroptosis suppressor gene signature was associated with poor prognosis of HCC patients who received sorafenib treatment (**Figure 1E**, **Figure S1C**). Therefore, the results suggest that suppressed ferroptotic activity is associated with sorafenib resistance.

### Gene-Silencing Activities of the Multi-siRNA Against GPX4 and DHODH

**Figure 2:**
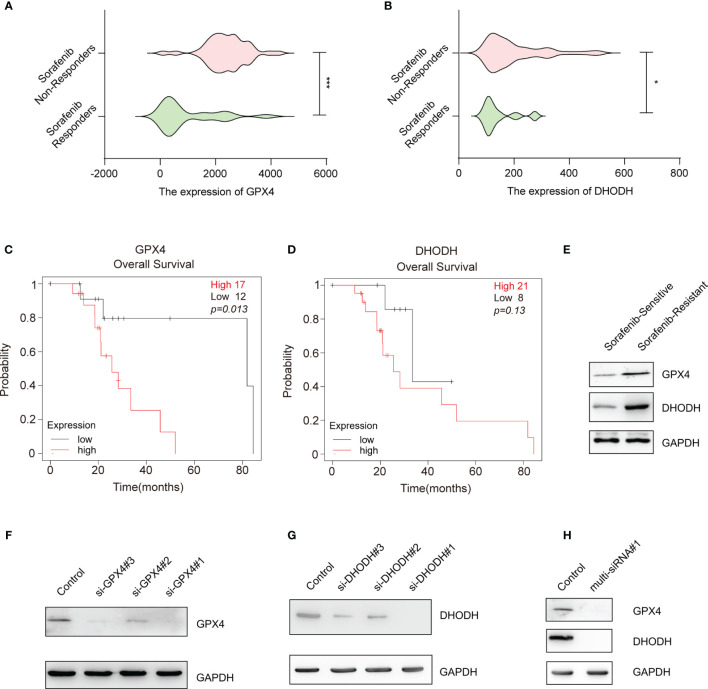
Gene-silencing activities of multi-siRNA against GPX4 and DHODH genes. **(A)** GPX4 expression in HCC tissues from sorafenib responders (n=21) or sorafenib nonresponders (n=43) (gse109211). **(B)** That of DHODH in the HCC tissues of sorafenib responders (n=21) or sorafenib nonresponders (n=43) (gse109211). **(C)** Kaplan–Meier overall survival analysis of the GPX4 gene in HCC patients who received sorafenib treatment. **(D)** Kaplan–Meier overall survival analysis of the DHODH gene in HCC patients who received sorafenib treatment. **(E)** Western blotting for assessment of the protein levels of DHODH and GPX4 in sorafenib-sensitive HepG-2 or sorafenib-resistant HepG-2 (HepG-2R) cells. **(F)** Western blotting was conducted to detect GPX4 expression in HepG-2R cells transfected with scramble or GPX4 siRNAs. **(G)** Western blotting was conducted to detect DHODH expression in HepG-2R cells transfected with scramble or DHODH siRNAs. **(H)** HepG-2R cells were transfected with scramble siRNA or multi-siRNA#1 containing the si-GPX4#1 and si-DHODH#1 target sequences, and Western blotting was conducted to detect GPX4 and DHODH expression. **(A, B)** Unpaired *t*-test. **(C, D)** Log-rank test. *p <0.05, ***p <0.001.

The above data suggest that silencing key ferroptosis suppressor genes increases the therapeutic effect of sorafenib. Among the ferroptosis suppressor genes, *GPX4* and DHODH can directly remove the dangerous products of iron-dependent lipid peroxidation and consequently protect the cell membrane from damage; when *GPX4* and DHODH expression and/or function are dysregulated, ferroptosis ensues (16, 17). Moreover, we found that the expression of GPX4 and DHODH was upregulated in sorafenib-resistant patients (**Figures 2A, B**) and associated with poor prognosis of HCC patients who received sorafenib treatment (**Figures 2C, D**). Meanwhile, GPX4 and DHODH expression was upregulated in sorafenib-resistant HCC cell line (HepG-2R) (**Figure 2E**). In light of these findings, we sought to interfere with the expression and function of these two genes at the same time. First, siRNAs targeting the human *GPX4* gene and human *DHODH* gene were designed and screened to determine their effectiveness. As shown in **Figures 2F, G** and **Figures S2A, B**, si-GPX4#1 and si-DHODH#1 were the most effective siRNAs in knocking down their respective proteins. Then, we designed a multi-siRNA containing the sequences corresponding to si-GPX4#1 and si-DHODH#1; the sequence “AUUGCAC” was used as a linker to connect si-GPX4#1 to si-DHODH#1. The results showed that this multi-siRNA could simultaneously suppress GPX4 and DHODH expression (**Figure 2H**).

### Preparation and Characterization of SP94-Lamp2b-RRM Fusion Protein-Engineered Exosomes

**Figure 3:**
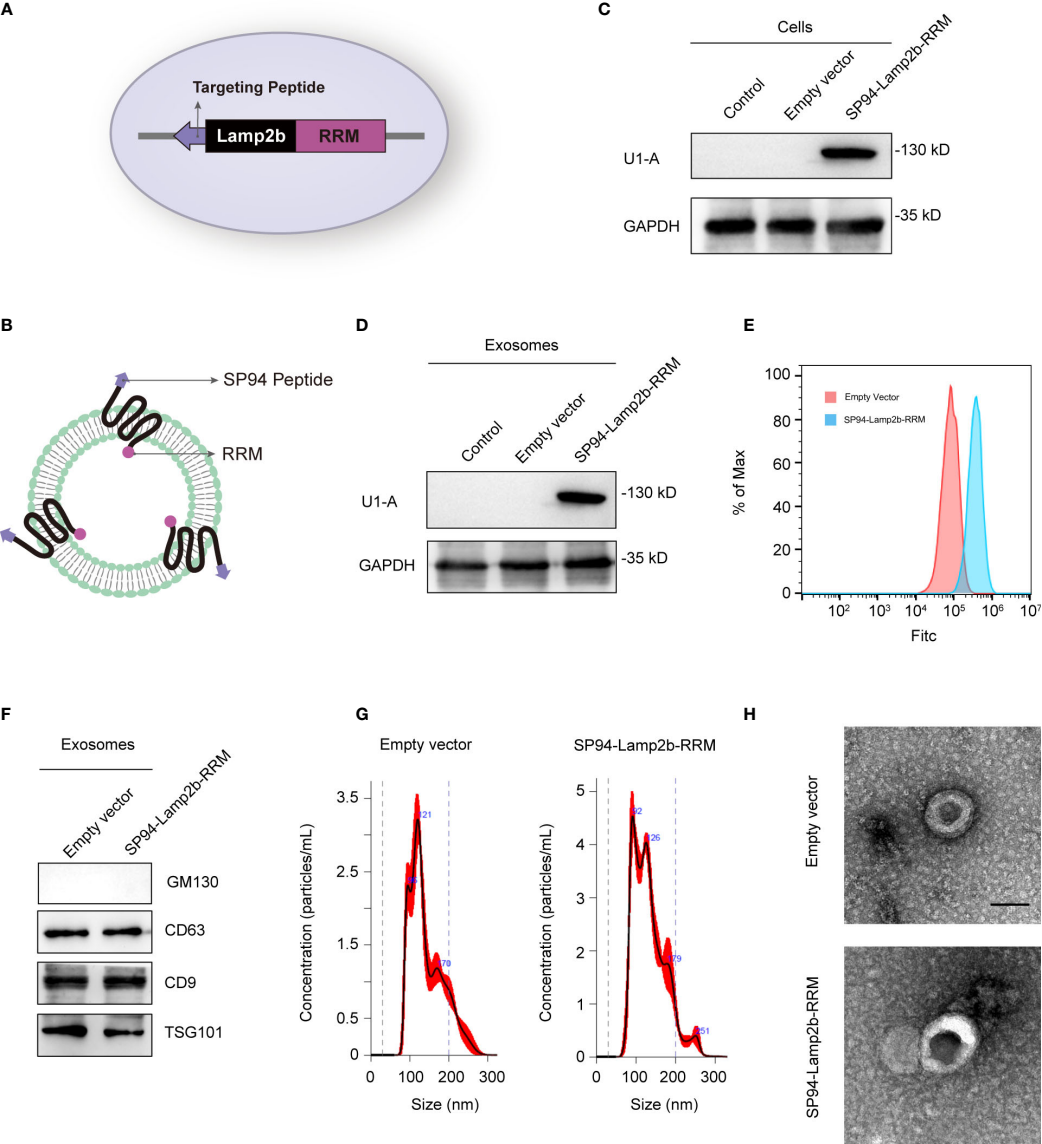
Preparation and characterization of SP94-Lamp2b-RRM fusion protein-engineered exosomes. **(A)** Schematic representation of the hepatoma-cell-targeting peptide SP94 and the RRM of U1-A fused to the N-terminus and C-terminus of Lamp2b. **(B)** Schematic representation of the structure of the SP94-Lamp2b-RRM fusion protein and the engineered exosomes. **(C)** Western blot analysis of U1-A expression in HEK-293T cells transfected with empty vector or SP94-Lamp2b-RRM plasmids. **(D)** U1-A expression in exosomes derived from HEK-293T cells transfected with empty vector or SP94-Lamp2b-RRM plasmids. **(E)** Equal amount of DiO-labeled exosomes were incubated with 1*10^6^ immobilized HepG-2 cells for 1h. After washing, unbound exosomes were removed. HepG-2 cells were used for flow cytometry after trypsinization. The binding of different exosomes to HepG-2 cells was then quantitated by flow cytometry (FITC channel). **(F)** Western blot analysis of the exosome markers CD63, CD9 and TSG101, and the exclusive exosome marker GM130 on exosomes derived from HEK-293T cells transfected with different plasmids. **(G)** Nanoparticle tracking analysis shows the size distribution of exosomes derived from HEK-293T cells transfected with different plasmids. **(H)** Representative TEM images of exosomes derived from control or SP94-Lamp2b-RRM plasmid-transfected HEK-293T cells (scale bar, 100 nm).

Exosomes have been considered a good siRNA delivery vehicle to reduce drug resistance (18). However, low cargo encapsulation efficiency and the lack of cell-type specific targeting remain major hurdles for their potential clinical application. Recently, some RNA-binding proteins have been shown to significantly promote exosomal miRNA cargo loading *via* interaction with common short sequence motifs present in miRNAs (19). Therefore, we hypothesized that fusion of specific RNA-binding proteins with the exosomal membrane proteins can increase the loading efficiency of the desired RNAs. We fused the N-terminal RNA recognition motif (RRM) of U1-A with the C-terminus of Lamp2b (exosomal surface protein) (**Figures 3A, B**). U1-A can bind the highly conserved sequence “AUUGCAC” in RNA *via* its N-terminal RRM (20). SP94 (protein sequence, SFSIIHTPILPL), is a novel peptide that has been reported to specifically bind to HCC cells (21). Therefore, we fused SP94 with the N-terminus of Lamp2b to enhance the tumor-targeting ability of the engineered exosomes (**Figures 3A, B**). The recombinant vector produced abundant expression of the fusion protein in transfected cells (**Figure 3C**). As the engineered SP94-Lamp2b-RRM fusion protein could express on exosomes (**Figure 3D**), we analyzed the binding ability of these exosomes to HCC cells. The results showed that exosomes derived from HEK-293T cells transfected with SP94-Lamp2b-RRM could bind strongly to HCC cells **(** **Figure 3E**), indicating that the SP94-Lamp2b-RRM fusion protein was incorporated into exosomes. In addition, specific exosome markers (CD63, TSG101 and CD9) were detected in the purified samples, while the Golgi marker GM130 was barely detectable (**Figure 3F**). Nanoparticle tracking analysis (NTA) revealed that the exosomes were similar in number and had a similar size distribution between the groups (**Figure 3G**). Transmission electron microscopy (TEM) confirmed that the purified exosomes exhibited a typical round-shaped vesicular morphology and were within the appropriate size range (**Figure 3H**).

### Efficient Therapeutic Cargo Encapsulation Into SP94-Lamp2b-RRM Fusion-Protein-Engineered Exosomes

**Figure 4:**
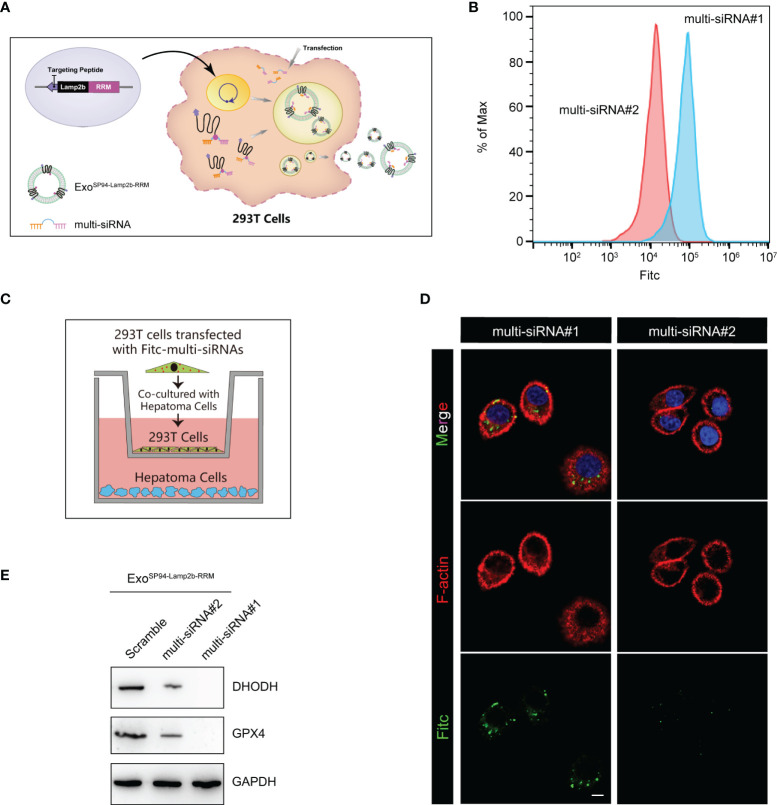
Efficient therapeutic cargo encapsulation into engineered exosomes expressing SP94-Lamp2b-RRM fusion protein. **(A)** Schematic diagram of the process of multi-siRNA encapsulation into exosomes *via* the SP94-Lamp2b-RRM fusion protein in HEK-293T cells. The SP94-Lamp2b-RRM fusion protein recruits multi-siRNA containing the sequence “AUUGCAC” to exosomes *via* RNA-RRM recognition. **(B)** HEK-293T cells transiently transfected with FITC-tagged multi-siRNA#1 or FITC-tagged multi-siRNA#2. Then, exosomes were collected for the flow cytometry assay (multi-siRNA#1, contains si-GPX4#1, si-DHODH#1 and the RRM binding sequence; multi-siRNA#2, contains si-GPX4#1 and si-DHODH#1 without the RRM binding sequence). **(C, D)** HEK-293T cells transiently transfected with FITC-tagged multi-siRNAs were cocultured with HepG-2 cells for 24 h. **(C)** Schematic illustration of HepG-2 and HEK-293T cell coculturing. **(D)** Representative confocal images of FITC (green) and F-actin (red) staining in HepG-2 cells. The nuclei were counterstained with DAPI (blue) (scale bar, 10 µm). **(E)** HepG-2 cells were treated with functionalized exosomes derived from HEK-293 T cells and containing SP94-Lamp2b-RRM, and Western blot assays were conducted to detect GPX4 and DHODH expression in HepG-2 cells. Scramble: 1*10^6^ HEK-293T cells were treated with the same amount of scramble siRNA; multi-siRNA#2: 1*10^6^ HEK-293T cells were treated with the same amount of multi-siRNA#2; multi-siRNA#1: 1*10^6^ HEK-293T cells were treated with the same amount of multi-siRNA#1. After 24 h, the exosomes were collected and added to HepG-2 cells.

Next, we explored whether the engineered SP94-Lamp2b-RRM fusion protein could promote RNA loading during exosome biogenesis. First, HEK-293T cells overexpressing the SP94-Lamp2b-RRM fusion protein were transfected with 100 nM corresponding FITC-tagged multi-siRNA (**Figure 4A**). Then, an equal number of exosomes from each group was evaluated by flow cytometry. The results showed that a higher amount of multi-siRNA containing the RRM recognition motif “AUUGCAC” could be sorted into exosomes compared to that of multi-siRNA#2 without the RRM binding sequence (**Figure 4B**). HEK-293T cells were then cocultured with HepG-2 cells (**Figure 4C**). Images from the confocal microscope showed a higher level of multi-siRNA containing the RRM “AUUGCAC” in HepG-2 cells (**Figure 4D**). Consistent with this finding, exosomes derived from HEK-293T cells transfected with the multi-siRNA containing the “AUUGCAC” sequence could significantly suppress the expression of GPX4 and DHODH in HCC cells **(** **Figure 4E**). These results suggest that the SP94-Lamp2b-RRM fusion protein promotes the exosomal loading of the “AUUGCAC” sequence linked to the multi-siRNA *via* RNA-protein interactions.

### SP94-Lamp2b-RRM-Functionalized Exosomes Could Overcome Sorafenib Resistance by Enhancing Sorafenib-Induced Ferroptosis in HCC Cells

**Figure 5:**
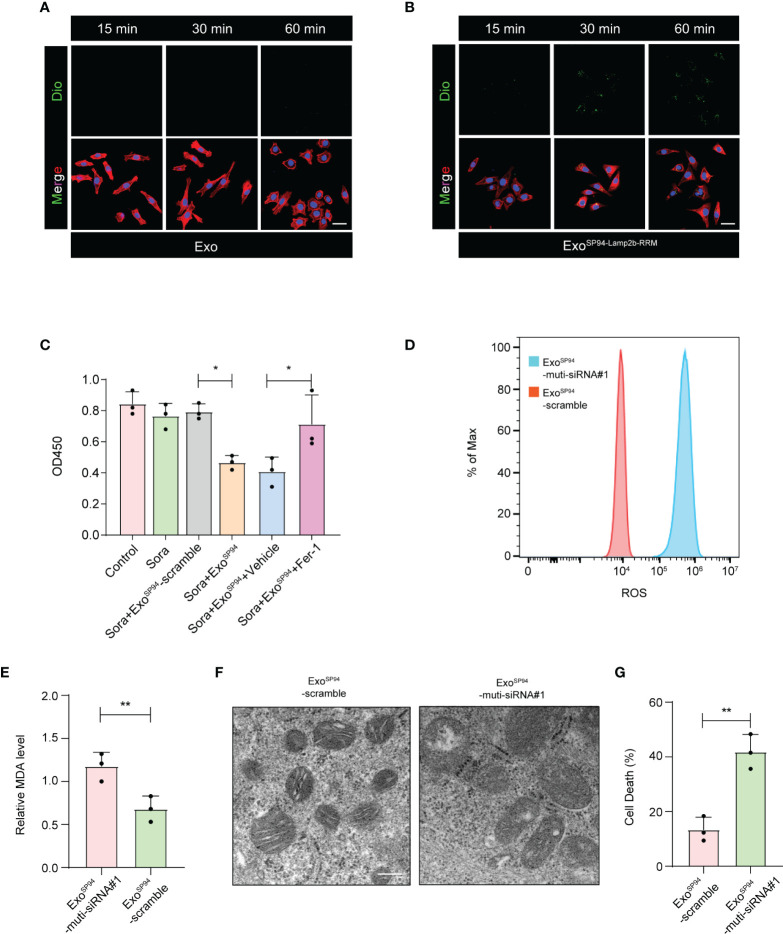
SP94-Lamp2b-RRM-functionalized exosomes could overcome sorafenib resistance by enhancing sorafenib-induced ferroptosis in HCC cells. **(A–B)** Dio labeled engineered exosomes or control exosomes were incubated with HCC cells for 15, 30 or 60 min at 37°C. Then unbound exosomes were removed by PBS washing. Exosome endocytosis was analyzed by confocal microscopy. The nuclei and F-actin were counterstained with DAPI (blue) and TRITC phalloidin (red), respectively, (scale bar, 50 µm). **(C)** Sorafenib-resistant HepG-2 cells treated with sorafenib and SP94-Lamp2b-RRM-functionalized exosomes containing scramble or multi-siRNA#1. Viability of sorafenib-resistant HepG-2 cells as measured by the CCK-8 assay. **(D)** Sorafenib-resistant HepG-2 cells were treated with sorafenib in combination with SP94-Lamp2b-RRM-functionalized exosomes containing scramble or multi-siRNA#1, and the ROS production was assessed by DCFH-DA staining followed by flow cytometry. **(E)** MDA levels of sorafenib-resistant HepG-2 cells treated with sorafenib and SP94-Lamp2b-RRM-functionalized exosomes containing scramble or multi-siRNA#1. **(F)** TEM images of mitochondria in sorafenib-resistant HepG-2 cells cotreated with sorafenib and SP94-Lamp2b-RRM-functionalized exosomes containing scramble or multi-siRNA#1. **(G)** Sorafenib-resistant HepG-2 cells were treated with sorafenib and SP94-Lamp2b-RRM-functionalized exosomes containing scramble or multi-siRNA#1. Cell death was determined by PI staining coupled with flow cytometry. **(C, E, G)** The data are shown as the means ± S.E.M. **(C)** ANOVA with Dunnett’s *t*-test. **(E, G)** Unpaired *t*-test. *p < 0.05, **p < 0.01.

To investigate whether Exo^SP94-Lamp2b-RRM^ can bind to HCC cells, blank exosomes or Exo^SP94-Lamp2b-RRM^ were labeled with DiO and added to sorafenib-resistant HepG-2 cell cultures. Confocal assay showed that green fluorescence appeared within 15 min and intensified over time (up to 60 min) in the SP94-Lamp2b-RRM-functionalized exosomes treated group. By contrast, relatively low level of green fluorescence was observed in control exosomes treated cells up to 60 min, confirming the targeting ability of SP94-Lamp2b-RRM-functionalized exosomes to HCC cells (**Figures 5A, B**). Then, we treated sorafenib-resistant HepG-2 cells with exosomes (Exo^SP94-Lamp2b-RRM^-multi-siRNA#1). The results showed that Exo^SP94-Lamp2b-RRM^-multi-siRNA#1 could significantly enhance the effect of sorafenib (**Figure 5C**). Then, we further measured ROS and lipid peroxidation levels, which are the primary drivers of ferroptosis. The results showed that treatment with Exo^SP94-Lamp2b-RRM^-multi-siRNA#1 significantly increased ROS and MDA levels in sorafenib-resistant cells (**Figures 5D, E**). In addition to biochemical analyses, TEM was used to observe morphological changes. Exo^SP94-Lamp2b-RRM^-multi-siRNA#1-treated cells displayed diminished or vanished mitochondrial cristae and condensed mitochondrial membrane densities compared to Exo^SP94-Lamp2b-RRM^-scramble-treated cells (**Figure 5F**). Moreover, we also found that Exo^SP94-Lamp2b-RRM^-multi-siRNA#1 could enhance sorafenib-induced ferroptotic cell death (**Figure 5G**). Taken together, these results suggested that Exo^SP94-Lamp2b-RRM^-multi-siRNA#1 could enhance sorafenib-induced ferroptosis in HCC cells by silencing the expression of GPX4 and DHODH.

### SP94-Lamp2b-RRM-Functionalized Exosomes Could Efficiently Target Hepatocellular Carcinoma *In Vivo*

**Figure 6:**
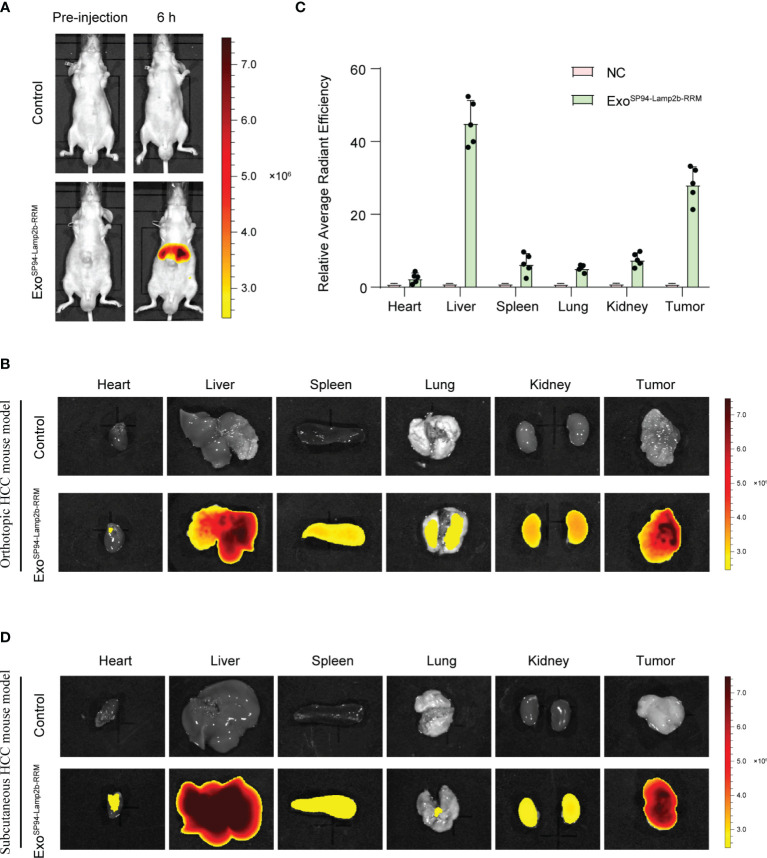
SP94-Lamp2b-RRM-functionalized exosomes could efficiently target HCC *in vivo.* ***(*A–C*)*** Sorafenib-resistant HepG-2 cells were orthotopically inoculated into the left liver lobes of nude mice. SP94-Lamp2b-RRM-functionalized exosomes (100 μg) were injected into nude mice *via* tail vein. **(A)** Mice were evaluated with a fluorescent live imaging system 6 h after injection. **(B)** Representative *ex vivo* fluorescence images of DiR-labeled exosomes in the main organs and HCC tissues. **(C)** Relative fluorescence intensity of DiR-labeled exosomes in different organs and liver cancer tissues. **(D)** Sorafenib-resistant HepG-2 cells were subcutaneously inoculated on the left backs of mice. SP94-Lamp2b-RRM-functionalized exosomes were loaded with DiR, and 100 μg of exosomes was injected into mice *via* tail vein. Different organs and tumors were harvested to conduct fluorescence imaging. **(C)** The data are shown as the means ± S.E. M; n=5.

To confirm whether SP94-Lamp2b-RRM-functionalized exosomes could target HCC *in vivo*, we tracked the distribution of DiR-labeled exosomes. First, we established an orthotopic liver injection model and observed that SP94-Lamp2b-RRM-functionalized exosomes were mainly distributed to the liver (**Figures 6A–C**). Meanwhile, SP94-Lamp2b-RRM-functionalized exosomes could target HCC (**Figures 6A–C**). Next, sorafenib-resistant HepG-2 cells were subcutaneously inoculated into the left backs of mice. In the subcutaneous HCC model, we found that SP94-Lamp2b-RRM-functionalized exosomes were mainly distributed in the liver and subcutaneous tumor tissues (**Figure 6D**, **Figure S3A**). The results from the different HCC models suggest that the SP94 targeting peptide dramatically enhances the ability of exosomes to bind HCC cells and tissues.

### Therapeutic Effects of Cotreatment With SP94-Lamp2b-RRM-Functionalized Exosomes and Sorafenib in an HCC Mouse Model

**Figure 7:**
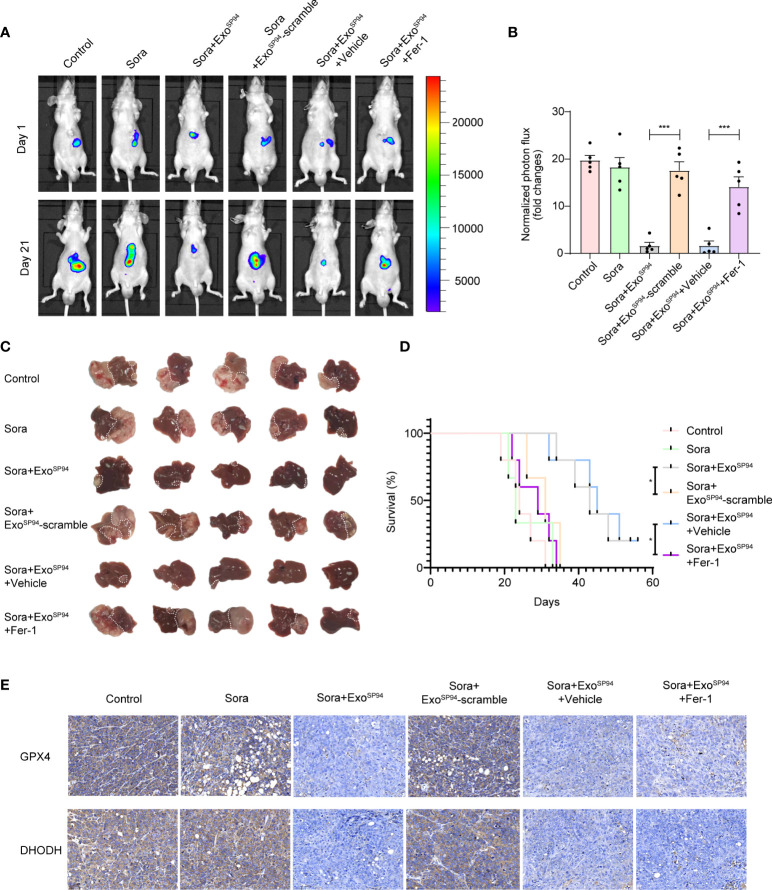
Therapeutic effects of SP94-Lamp2b-RRM-functionalized exosomes and sorafenib in an HCC mouse model. Sorafenib-resistant HepG-2 cells were orthotopically injected into the left liver lobes of nude mice. Mice with a similar tumor size were used. Control: saline; Sora: sorafenib; Sora+Exo^SP94^: sorafenib plus SP94-Lamp2b-RRM-functionalized exosomes containing multi-siRNA#1; Sora+Exo^SP94^-scramble: sorafenib plus SP94-Lamp2b-RRM-functionalized exosomes containing scramble siRNA; Sora+Exo^SP94^+Vehicle: sorafenib plus SP94-Lamp2b-RRM-functionalized exosomes containing multi-siRNA#1 in vehicle; Sora+Exo^SP94^+Fer-1: sorafenib plus SP94-Lamp2b-RRM-functionalized exosomes in multi-siRNA#1 combined with ferrostatin-1. Tumor growth was measured with an IVIS imaging system. **(A)** Representative tumor imaging on days 1 and 21 after the first treatment. **(B)** The normalized photon flux at day 21 was analyzed (n=5). **(C)** Image of resected xenografts of nude mice injected intrahepatically with Sorafenib-resistant HepG-2 cells. **(D)** Survival of mice from different groups (n=5). **(E)** Representative immunohistochemical staining for GPX4 and DHODH in HCC tissues from different groups (scale bar, 25 μm). **(B)** The data are shown as the means ± S.E.M. ANOVA with Dunnett’s *t*-test. **(D)** Log-rank test. ***p < 0.001.

Next, we explored the therapeutic effect of SP94-Lamp2b-RRM-functionalized exosomes combined with sorafenib *in vivo*. While the tumors of mice treated with sorafenib and Exo^SP94-Lamp2b-RRM^ containing scramble multi-siRNA grew rapidly, those of mice treated with sorafenib and Exo^SP94-Lamp2b-RRM^ containing multi-siRNA#1 were significantly reduced after 21 days of treatment (**Figures 7A–C**). However, the effect of sorafenib and Exo^SP94-Lamp2b-RRM^ containing multi-siRNA#1 on inhibiting tumor growth was diminished when cells were treated with the ferroptosis inhibitor ferrostatin-1 (**Figures 7A–C**). Similarly, mice from the sorafenib and Exo^SP94-Lamp2b-RRM^ containing multi-siRNA#1 cotreatment group had a longer life expectancy than did mice from the sorafenib and Exo^SP94-Lamp2b-RRM^ containing scramble multi-siRNA treatment group (**Figure 7D**). To examine the knockdown efficiency in each group, GPX4 and DHODH expression in primary tumor lesions was investigated by immunohistochemistry. The results showed that multi-siRNA#1 could significantly suppress GPX4 and DHODH expression *in vivo* (**Figure 7E**). Taken together, these results suggested that Exo^SP94-Lamp2b-RRM^-multi-siRNA#1 could overcome sorafenib resistance *in vivo* by silencing the ferroptosis suppressor genes GPX4 and DHODH.

### Systemic Toxicity Evaluation

**Figure 8:**
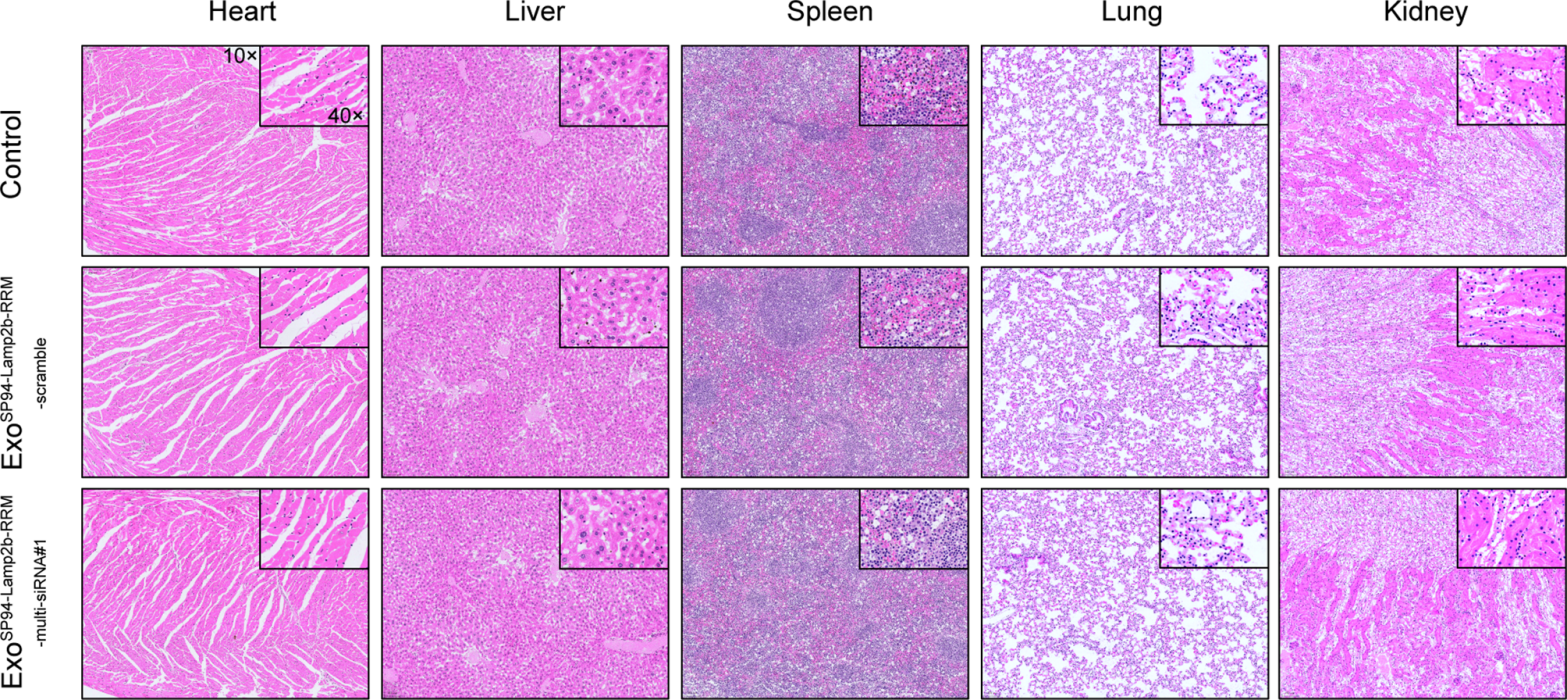
Systemic toxicity evaluation. One hundred micrograms of SP94-Lamp2b-RRM-functionalized exosomes were injected into nude mice *via* tail vein every 3 days for 21 days. Control: saline; Sora+Exo^SP94-Lamp2b-RRM^-scramble: sorafenib plus SP94-Lamp2b-RRM-functionalized exosomes containing scramble siRNA; Sora+Exo^SP94-Lamp2b-RRM^-multi-siRNA#1: sorafenib plus SP94-Lamp2b-RRM-functionalized exosomes containing multi-siRNA#1; Representative hematoxylin-eosin (H&E) staining of the main organs from different treatment groups (scale bar, 100 μm).

To evaluate the systemic toxicity of Exo^SP94-Lamp2b-RRM^, exosomes containing scramble or multi-siRNA#1 were injected into nude mice *via* tail vein. There was no significant difference in the body weights of mice in the two groups (**Figure S4A**). In addition, compared to the control group, the Exo^SP94-Lamp2b-RRM^ group showed relatively normal tissue structure and morphology (**Figure 8**). These results indicate that there was no obvious adverse effect after treatment with Exo^SP94-Lamp2b-RRM^.

## Discussion

Our study demonstrated that sorafenib could induce ferroptosis in HCC cells, which is in line with the literature stating that the anticancer activity of sorafenib mainly relies on the induction of ferroptosis. What’s more, we found that the expression of ferroptosis suppressor genes, especially GPX4 and DHODH, was enriched in sorafenib-resistant HCC cells and patient samples, which suggests that suppressed ferroptotic activity is associated with compromised therapeutic efficiency of sorafenib. Thus, precise elimination of ferroptosis suppressor genes might be a new promising strategy to enhance sorafenib efficacy and improve patient prognosis.

Ferroptosis is a form of regulated cell death that is induced by excessive lipid peroxide accumulation in cellular membranes (22–24). Cells have developed at least two major defensive arms to detoxify lipid peroxides: GPX4 and DHODH. Consequently, disabling one arm forces cells to be more dependent on the other. For example, inhibition of either DHODH or GPX4 alone did not induce ferroptosis in HT-1080 cells, which is likely due to the relatively high endogenous expression of GPX4 and DHODH (25); interestingly, a GPX4 inhibitor combined with a DHODH inhibitor could synergistically suppress HT-1080 tumor growth. Similarly, in our study, the sorafenib-resistant cells and tissue samples exhibited high levels of GPX4 and DHODH expression. Therefore, disabling both arms might better enhance sorafenib-induced ferroptosis. Here, we created a multi-siRNA that can simultaneously knock down GPX4 and DHODH *in vitro* and *in vivo*. To our knowledge, this is the first ferroptosis inducer that can directly target two genes. Additionally, this novel construct provides a new approach for the clinical treatment of sorafenib-resistant cancer.

Because of their intrinsic nature, exosomes are biocompatible with the host immune system and have an innate ability to protect and transport small RNAs and other critical molecules across biological barriers *in vivo*; they have been increasingly recognized as promising vehicles to deliver siRNA *in vivo* (26, 27). At present, the most common way to load nucleic acids into exosomes is *via* electroporation or direct encapsulation in donor cells (28); however, these methods have relatively low loading efficiencies. Meanwhile, *in vitro* loading of naked siRNAs into exosomes by electroporation could cause extensive siRNA aggregation and significantly reduce the level of bioactive siRNAs (29). Thus, there is an urgent need to develop a strategy for efficient loading of nucleic acids, especially siRNA. Recently, research conducted by our laboratory and others have observed that some RNA-binding proteins can efficiently sort miRNAs and other RNAs into exosomes *via* protein-RNA interactions (30, 31). In light of these data, we hypothesized that fusion of an exosomal membrane protein with a specific RNA-binding protein would increase the loading efficiency of the siRNA of interest; therefore, we fused the RNA recognition motif of U1-A with the C-terminus of Lamp2b, which interacts with the sequence “AUUGCAC” in the target RNA with a relatively high affinity. Then, a multi-siRNA was engineered to harbor the consensus “AUUGCAC” sequence. The preliminary data here indicate that the fusion protein helps sort multiple siRNAs containing the “AUUGCAC” sequence into exosomes during exosome biogenesis, especially when the RNA of interest is exogenously overexpressed. It is worth noting that due to the limitation of the present siRNA detection technology, the biological loading rate of multi-siRNA cannot be accurately detected in live cells and exosomes. Here, in the preparation of functionalized exosomes, saturated amount of multi-siRNAs were transfected into cells to ensure that engineered exosomes were loaded with sufficient multi-siRNAs. Further experiments also confirmed that a large amount of multi-siRNAs could be effectively encapsulated into engineered exosomes and delivered to HCC cells, thus inducing down-regulation of GPX4 and DHODH. Our study established a novel strategy to efficiently load therapeutic multi-siRNA cargos into exosomes.

Currently, the poor solubility and potential off-target toxicity to normal cells and tissues preclude the systematic use of traditional ferroptosis inducers *in vivo* (32–34). Nanoscale exosomes are considered a good choice as a drug vehicle because exosomes could be engineered toward better targeting specificity *via* exosome surface protein modifications (35–39). For example, exosomes expressing the αγ integrin-specific iRGD peptide fused to LAMP-2b efficiently delivered doxorubicin to integrin-positive breast cancer cells *in vitro* and *in vivo* (40). Here, an HCC-specific targeting peptide, SP94, was fused to the extracellular domain of LAMP-2B at the N-terminus (21). Our data show that functionalized exosomes (Exo^SP94-lamp2b-RRM^-multi-siRNA) can selectively deliver therapeutic cargos to human HCC cells with no obvious adverse effects. This is the first report of tumor-targeted exosome delivery of ferroptosis inducers, which might open a new avenue for the systematic use of ferroptosis inducers to treat cancer.

In summary, this study firstly identified that ferroptosis suppressor genes GPX4 and DHODH were enriched in sorafenib-resistant HCC and associated with compromised therapeutic efficiency of sorafenib. In addition, we described the use of multi-siRNA to enhance sorafenib-induced ferroptosis by simultaneously knocking down GPX4 and DHODH. More importantly, we designed HCC-targeted engineered exosomes (Exo^SP94-Lamp2b-RRM^) to deliver multi-siRNA, thus overcoming acquired resistance to sorafenib from the perspective of ferroptosis.

## Data Availability Statement

The datasets presented in this study can be found in online repositories. The names of the repository/repositories and accession number(s) can be found in the article/**Supplementary Material**.

## Ethics Statement

The animal study was reviewed and approved by Animal Care and Use Committee of Fourth Military Medical University.

## Author Contributions

XL, QY, and RZ contributed equally to this work. YG, CZ, and YZ contributed to project conceptualization and supervision. YG, XL, QY, CL, XG, RZ, and KZ contributed to the investigation. WQZ, SW, QH, WL, and ML contributed to the data curation; YG and XL contributed to writing the original draft; YG, CZ, YZ, WZ, and XL contributed to writing, reviewing, and editing the manuscript; and YG, CZ, and SW contributed to funding acquisition. All authors contributed to the article and approved the submitted version.

## Funding

This work was financially supported by grants from the Hong Kong Scholar Program; the National Natural Science Foundation of China (NSFC), NO. 81902678, 81802632, 82072910; the Key Research and Development Program of Shaanxi Province (NO. 2017ZDCXL-SF-01-03); and the Young Elite Scientist Sponsorship Program by CAST (No. YESS20210177).

## Conflict of Interest

The authors declare that the research was conducted in the absence of any commercial or financial relationships that could be construed as a potential conflict of interest.

## Publisher’s Note

All claims expressed in this article are solely those of the authors and do not necessarily represent those of their affiliated organizations, or those of the publisher, the editors and the reviewers. Any product that may be evaluated in this article, or claim that may be made by its manufacturer, is not guaranteed or endorsed by the publisher.

## References

[1] Siegel RL Miller KD Jemal A. Cancer Statistics, 2018. CA Cancer J Clin (2018) 68:7–30. doi: 10.3322/caac.21442 29313949

[2] Bray F Ferlay J Soerjomataram I Siegel RL Torre LA Jemal A. Global Cancer Statistics 2018: GLOBOCAN Estimates of Incidence and Mortality Worldwide for 36 Cancers in 185 Countries. CA Cancer J Clin (2018) 68:394–424. doi: 10.3322/caac.21492 30207593

[3] Yang JD Hainaut P Gores GJ Amadou A Plymoth A Roberts LR. A Global View of Hepatocellular Carcinoma: Trends, Risk, Prevention and Management. Nat Rev Gastroenterol Hepatol (2019) 16:589–604. doi: 10.1038/s41575-019-0186-y 31439937 PMC6813818

[4] Cheng AL Kang YK Chen Z Tsao CJ Qin S Kim JS. Efficacy and Safety of Sorafenib in Patients in the Asia-Pacific Region With Advanced Hepatocellular Carcinoma: A Phase III Randomised, Double-Blind, Placebo-Controlled Trial. Lancet Oncol (2009) 10:25–34. doi: 10.1016/S1470-2045(08)70285-7 19095497

[5] Chen X Kang R Kroemer G Tang D. Broadening Horizons: The Role of Ferroptosis in Cancer. Nat Rev Clin Oncol (2021) 18:280–96. doi: 10.1038/s41571-020-00462-0 33514910

[6] Wang Q Bin C Xue Q Gao Q Huang A Wang K. GSTZ1 Sensitizes Hepatocellular Carcinoma Cells to Sorafenib-Induced Ferroptosis *via* Inhibition of NRF2/GPX4 Axis. Cell Death Dis (2021) 12:426. doi: 10.1038/s41419-021-03718-4 33931597 PMC8087704

[7] Su Y Zhao B Zhou L Zhang Z Shen Y Lv H. Ferroptosis, a Novel Pharmacological Mechanism of Anti-Cancer Drugs. Cancer Lett (2020) 483:127–36. doi: 10.1016/j.canlet.2020.02.015 32067993

[8] Sun X Ou Z Chen R Niu X Chen D Kang R. Activation of the P62-Keap1-NRF2 Pathway Protects Against Ferroptosis in Hepatocellular Carcinoma Cells. Hepatology (2016) 63:173–84. doi: 10.1002/hep.28251 PMC4688087 26403645

[9] Lachaier E Louandre C Godin C Saidak Z Baert M Diouf M. Sorafenib Induces Ferroptosis in Human Cancer Cell Lines Originating From Different Solid Tumors. Anticancer Res (2014) 34:6417–22. 25368241

[10] Dixon SJ Patel DN Welsch M Skouta R Lee ED Hayano M. Pharmacological Inhibition of Cystine-Glutamate Exchange Induces Endoplasmic Reticulum Stress and Ferroptosis. Elife (2014) 3:e2523. doi: 10.7554/eLife.02523 PMC4054777 24844246

[11] Coriat R Nicco C Chereau C Mir O Alexandre J Ropert S. Sorafenib-Induced Hepatocellular Carcinoma Cell Death Depends on Reactive Oxygen Species Production *In Vitro* and *In Vivo* . Mol Cancer Ther (2012) 11:2284–93. doi: 10.1158/1535-7163.MCT-12-0093 22902857

[12] Dietrich P Koch A Fritz V Hartmann A Bosserhoff AK Hellerbrand C. Wild Type Kirsten Rat Sarcoma is a Novel microRNA-622-Regulated Therapeutic Target for Hepatocellular Carcinoma and Contributes to Sorafenib Resistance. Gut (2018) 67:1328–41. doi: 10.1136/gutjnl-2017-315402 29275358

[13] Gao Y Wang Z Hao Q Li W Xu Y Zhang J. Loss of ERalpha Induces Amoeboid-Like Migration of Breast Cancer Cells by Downregulating Vinculin. Nat Commun (2017) 8:14483. doi: 10.1038/ncomms14483 28266545 PMC5344302

[14] Dixon SJ Stockwell BR. The Role of Iron and Reactive Oxygen Species in Cell Death. Nat Chem Biol (2014) 10:9–17. doi: 10.1038/nchembio.1416 24346035

[15] Seibt TM Proneth B Conrad M. Role of GPX4 in Ferroptosis and its Pharmacological Implication. Free Radic Biol Med (2019) 133:144–52. doi: 10.1016/j.freeradbiomed.2018.09.014 30219704

[16] Feng H Stockwell BR. Unsolved Mysteries: How Does Lipid Peroxidation Cause Ferroptosis? PloS Biol (2018) 16:e2006203. doi: 10.1371/journal.pbio.2006203 29795546 PMC5991413

[17] Wang F Min J. DHODH Tangoing With GPX4 on the Ferroptotic Stage. Signal Transduct Target Ther (2021) 6:244. doi: 10.1038/s41392-021-00656-7 34145214 PMC8212586

[18] Herrmann IK Wood M Fuhrmann G. Extracellular Vesicles as a Next-Generation Drug Delivery Platform. Nat Nanotechnol (2021) 16:748–59. doi: 10.1038/s41565-021-00931-2 34211166

[19] Wozniak AL Adams A King KE Dunn W Christenson LK Hung WT. The RNA Binding Protein FMR1 Controls Selective Exosomal miRNA Cargo Loading During Inflammation. J Cell Biol (2020) 219:e201912074. doi: 10.1083/jcb.201912074 32970791 PMC7659717

[20] Bolduc F Turcotte MA Perreault JP. The Small Nuclear Ribonucleoprotein Polypeptide a (SNRPA) Binds to the G-Quadruplex of the BAG-1 5'utr. Biochimie (2020) 176:122–7. doi: 10.1016/j.biochi.2020.06.013 32629040

[21] Zhou T Liang X Wang P Hu Y Qi Y Jin Y. A Hepatocellular Carcinoma Targeting Nanostrategy With Hypoxia-Ameliorating and Photothermal Abilities That, Combined With Immunotherapy, Inhibits Metastasis and Recurrence. ACS Nano (2020) 14:12679–96. doi: 10.1021/acsnano.0c01453 32909732

[22] Garcia-Bermudez J Birsoy K. A Mitochondrial Gatekeeper That Helps Cells Escape Death by Ferroptosis. Nature (2021) 593:514–5. doi: 10.1038/d41586-021-01203-8 33981062

[23] Stockwell BR Friedmann AJ Bayir H Bush AI Conrad M Dixon SJ. Ferroptosis: A Regulated Cell Death Nexus Linking Metabolism, Redox Biology, and Disease. Cell (2017) 171:273–85. doi: 10.1016/j.cell.2017.09.021 PMC5685180 28985560

[24] Zhang Y Shi J Liu X Feng L Gong Z Koppula P. BAP1 Links Metabolic Regulation of Ferroptosis to Tumour Suppression. Nat Cell Biol (2018) 20:1181–92. doi: 10.1038/s41556-018-0178-0 PMC6170713 30202049

[25] Mao C Liu X Zhang Y Lei G Yan Y Lee H. DHODH-Mediated Ferroptosis Defence is a Targetable Vulnerability in Cancer. Nature (2021) 593:586–90. doi: 10.1038/s41586-021-03539-7 PMC8895686 33981038

[26] Fu Z Zhang X Zhou X Ur-Rehman U Yu M Liang H. *In Vivo* Self-Assembled Small RNAs as a New Generation of RNAi Therapeutics. Cell Res (2021) 31:631–48. doi: 10.1038/s41422-021-00491-z PMC8169669 33782530

[27] van Niel G D'Angelo G Raposo G. Shedding Light on the Cell Biology of Extracellular Vesicles. Nat Rev Mol Cell Biol (2018) 19:213–28. doi: 10.1038/nrm.2017.125 29339798

[28] Ohno S Takanashi M Sudo K Ueda S Ishikawa A Matsuyama N. Systemically Injected Exosomes Targeted to EGFR Deliver Antitumor microRNA to Breast Cancer Cells. Mol Ther (2013) 21:185–91. doi: 10.1038/mt.2012.180 PMC3538304 23032975

[29] Kooijmans S Stremersch S Braeckmans K de Smedt SC Hendrix A Wood M. Electroporation-Induced siRNA Precipitation Obscures the Efficiency of siRNA Loading Into Extracellular Vesicles. J Control Release (2013) 172:229–38. doi: 10.1016/j.jconrel.2013.08.014 23994516

[30] Gao Y Li X Zeng C Liu C Hao Q Li W. CD63(+) Cancer-Associated Fibroblasts Confer Tamoxifen Resistance to Breast Cancer Cells Through Exosomal miR-22. Adv Sci (Weinh). (2020) 7:2002518. doi: 10.1002/advs.202002518 33173749 PMC7610308

[31] Zhang H Deng T Liu R Ning T Yang H Liu D. CAF Secreted miR-522 Suppresses Ferroptosis and Promotes Acquired Chemo-Resistance in Gastric Cancer. Mol Cancer (2020) 19:43. doi: 10.1186/s12943-020-01168-8 32106859 PMC7045485

[32] Yu M Gai C Li Z Ding D Zheng J Zhang W. Targeted Exosome-Encapsulated Erastin Induced Ferroptosis in Triple Negative Breast Cancer Cells. Cancer Sci (2019) 110:3173–82. doi: 10.1111/cas.14181 PMC6778638 31464035

[33] Lu B Chen XB Ying MD He QJ Cao J Yang B. The Role of Ferroptosis in Cancer Development and Treatment Response. Front Pharmacol (2017) 8:992. doi: 10.3389/fphar.2017.00992 29375387 PMC5770584

[34] Stockwell BR Jiang X. The Chemistry and Biology of Ferroptosis. Cell Chem Biol (2020) 27:365–75. doi: 10.1016/j.chembiol.2020.03.013 PMC7204503 32294465

[35] Yi J Minikes AM Jiang X. Aiming at Cancer *In Vivo*: Ferroptosis-Inducer Delivered by Nanoparticles. Cell Chem Biol (2019) 26:621–2. doi: 10.1016/j.chembiol.2019.05.002 PMC6936732 31100261

[36] Terasawa K Tomabechi Y Ikeda M Ehara H Kukimoto-Niino M Wakiyama M. Lysosome-Associated Membrane Proteins-1 and -2 (LAMP-1 and LAMP-2) Assemble *via* Distinct Modes. Biochem Biophys Res Commun (2016) 479:489–95. doi: 10.1016/j.bbrc.2016.09.093 27663661

[37] Armstrong JP Holme MN Stevens MM. Re-Engineering Extracellular Vesicles as Smart Nanoscale Therapeutics. ACS Nano (2017) 11:69–83. doi: 10.1021/acsnano.6b07607 28068069 PMC5604727

[38] Yang J Wu S Hou L Zhu D Yin S Yang G. Therapeutic Effects of Simultaneous Delivery of Nerve Growth Factor mRNA and Protein *via* Exosomes on Cerebral Ischemia. Mol Ther Nucleic Acids (2020) 21:512–22. doi: 10.1016/j.omtn.2020.06.013 PMC7365960 32682291

[39] Kim H Yun N Mun D Kang JY Lee SH Park H. Cardiac-Specific Delivery by Cardiac Tissue-Targeting Peptide-Expressing Exosomes. Biochem Biophys Res Commun (2018) 499:803–8. doi: 10.1016/j.bbrc.2018.03.227 29621543

[40] Tian Y Li S Song J Ji T Zhu M Anderson GJ. A Doxorubicin Delivery Platform Using Engineered Natural Membrane Vesicle Exosomes for Targeted Tumor Therapy. Biomaterials (2014) 35:2383–90. doi: 10.1016/j.biomaterials.2013.11.083 24345736

