# Supplementary material for: Designer Exosomes for Targeted Delivery of a Novel Therapeutic Cargo to Enhance Sorafenib-Mediated Ferroptosis in Hepatocellular Carcinoma
Source: Front Oncol. 2022 Jun 24;12:898156. doi: 10.3389/fonc.2022.898156 (PMC9263838; doi:10.3389/fonc.2022.898156)
Supplement: Supplementary file 1 [file DataSheet_1.docx]

Supplementary Material

**1. Supplementary Figures
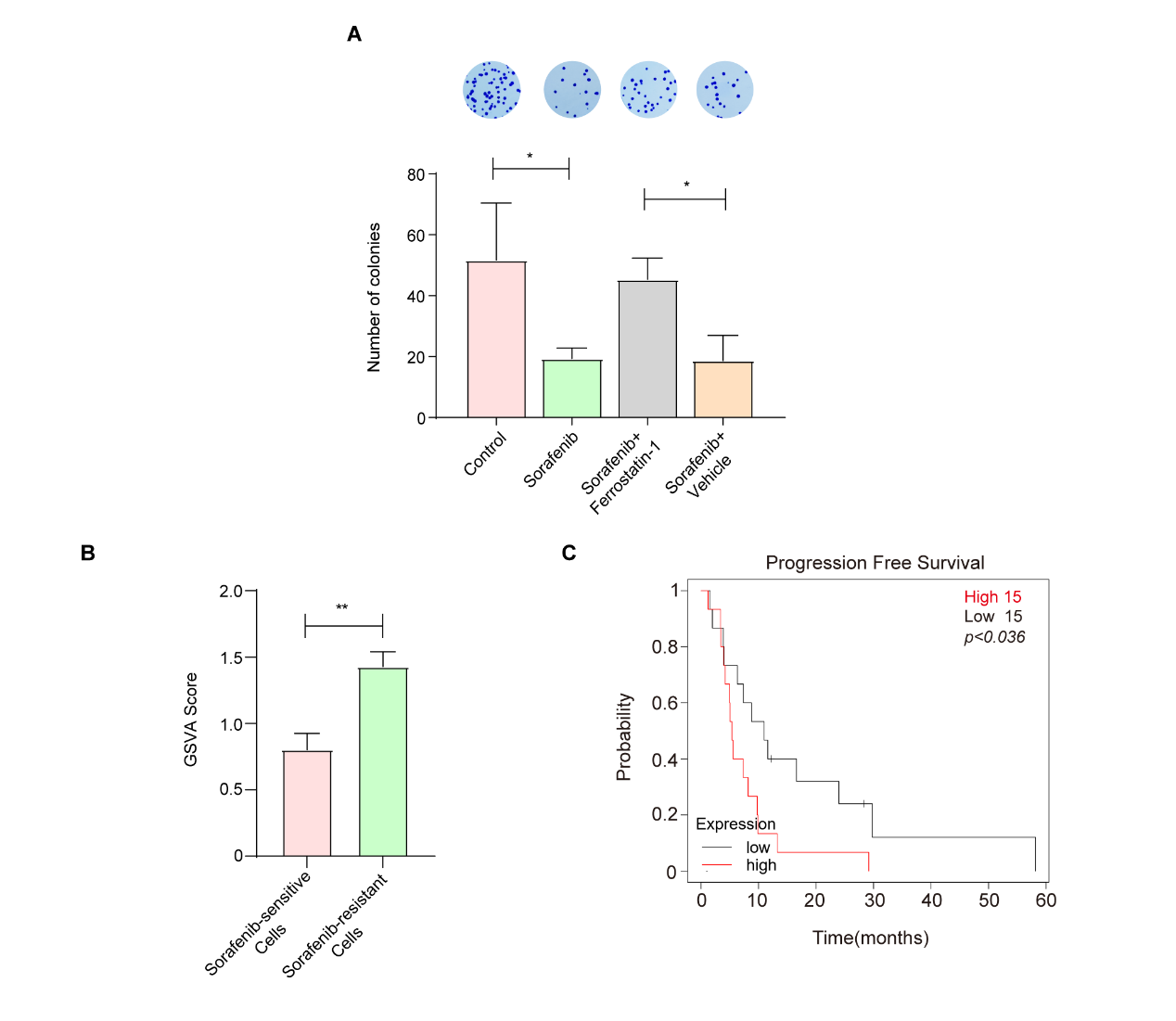
**

**Figure S1 Suppressed ferroptotic activity during sorafenib treatment is associated with compromised therapeutic efficiency. Related to figure 1.**

(**A**) Colony formation ability of HepG-2 cells from the control, sorafenib, sorafenib plus vehicle and sorafenib plus ferrostatin-1 groups. (**B**) GSVA was conducted to calculate the score for enrichment of ferroptosis suppressor genes. Hepatocellular carcinoma cells with sorafenib sensitivity or sorafenib resistance were obtained from the Gene Expression Omnibus (GSE176151). (**C**) Kaplan–Meier progression-free survival analysis of the ferroptosis suppressor gene signature in HCC patients who received sorafenib treatment. (**A, B**) The data are shown as the means ± S.E.M. (**A**) ANOVA with Dunnett’s *t*-test. (**B**) Unpaired *t*-test. (**C**) Log-rank test. **p* <0.05, ***p* <0.01.


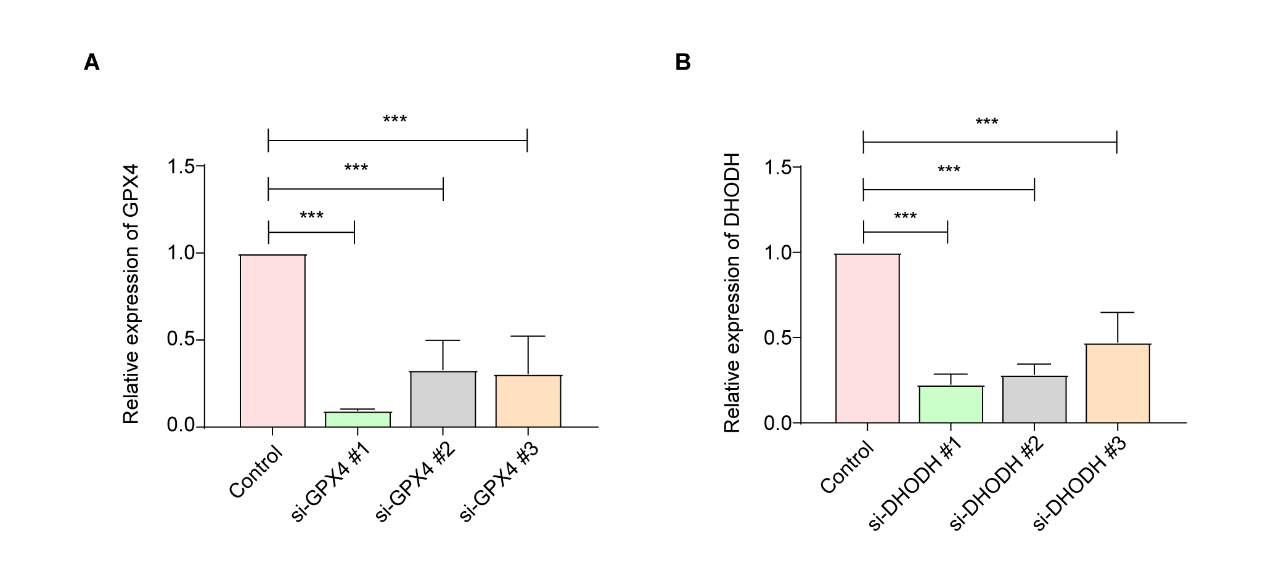


**Figure S2 Gene-silencing activities of multi-siRNA against GPX4 and DHODH genes. Related to figure 2.**

(**A**) Real-time PCR was conducted to detect GPX4 mRNA levels in HepG-2 cells transfected with scramble or GPX4 siRNAs. (**B**) Real-time PCR was conducted to detect the DHODH mRNA level in HepG-2 cells transfected with scramble or DHODH siRNAs. (**A, B**) The data are shown as the means ± S.E.M. ANOVA with Dunnett’s *t*-test. ****p* <0.001.


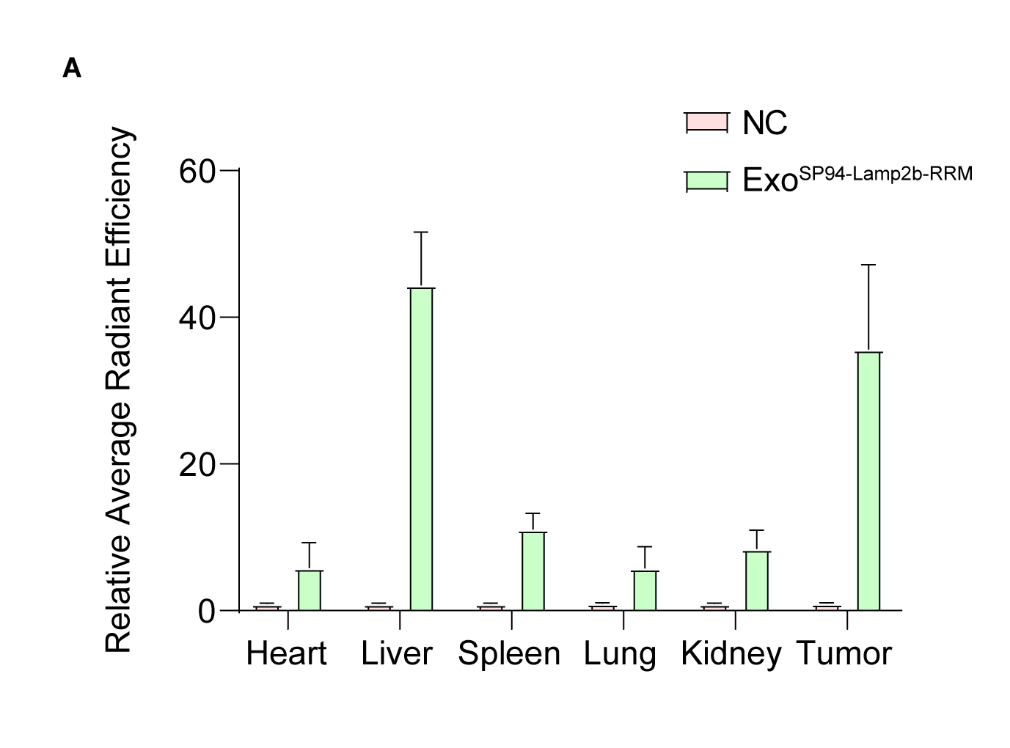


**Figure S3 SP94-Lamp2b-RRM-functionalized exosomes could efficiently target hepatocellular carcinoma *in vivo.* Related to figure 6.**

(**A**) Relative fluorescence intensity of DiR-labeled exosomes in different organs and tumors in Figure 6D.

**
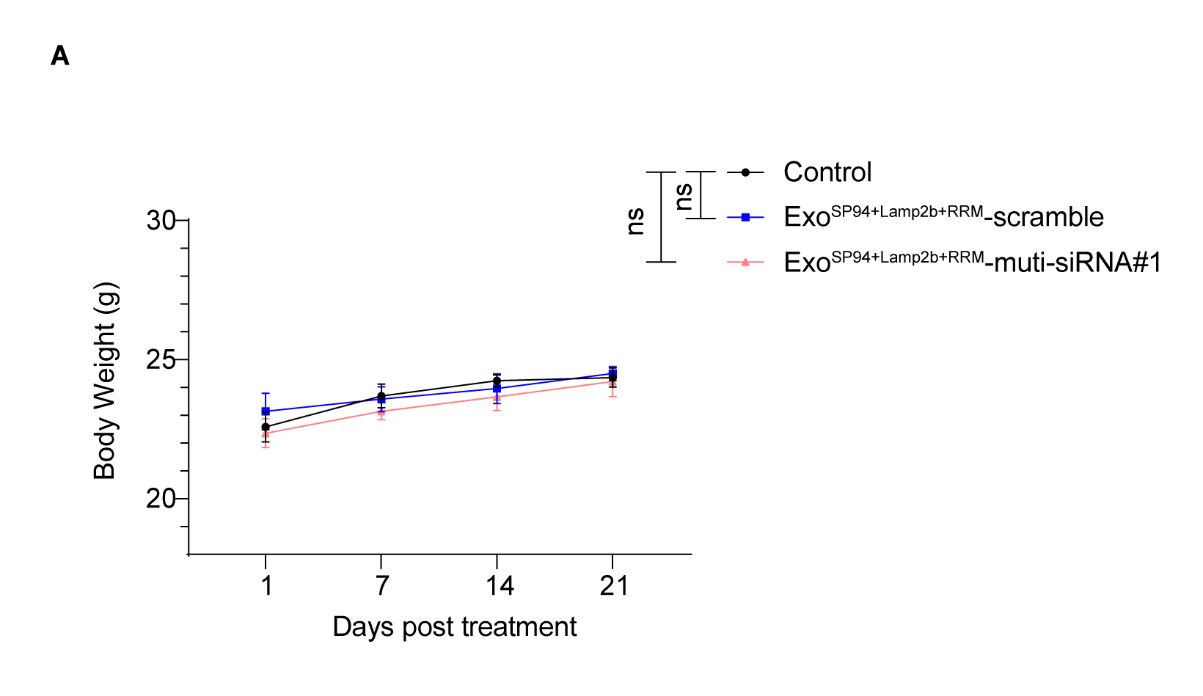
**

**Figure S4 Systemic toxicity evaluation. Related to figure 8.**

**(A)** The body weight of each mouse was recorded every seven days. (**A**) The data are shown as the means ± S.E.M. ANOVA with Dunnett’s *t*-test. ns *p* >0.05.

**2. Supplementary Methods**

**2.1 Cell proliferation assay.**

The viability of cells was measured by Cell Counting Kit-8 (CCK-8) solution (7sea biotech, China). Briefly, cancer cells were seeded on 96-well plates (Corning, USA) and incubated at 37 °C overnight. The CCK-8 reagents were then added to a subset of wells. After the cells were incubated for 2 h at 37 °C, we quantified the absorbance at 450 nm using a microplate reader (Bio–Rad, USA). Each group was made in quintuplicate.

**2.2 Western blot analysis**

The cells were lysed on ice, according to the instructions. Protease inhibitor cocktail (MedChem Express, USA) was used in cell lysates to increase protein stability. After SDS–PAGE, the proteins were transferred to PVDF membranes (0.22 µm, Invitrogen) using a Bio–Rad Semi-Dry Electrophoretic Transfer Cell. Western blot analyses were performed using corresponding specific antibodies, followed by HRP-conjugated IgG antibody. Enhanced chemiluminescence against HRP was used for the visualization of immunoreactive proteins.

**2.3 qRT–PCR**

Total RNA was isolated from cultured cells with RNAiso Plus (Takara, Dalian, China), and cDNA was synthesized with the PrimeScript RT Reagent Kit (Vazyme, Nanjing, China). Then, cDNA and SYBR Green Ex Taq (Vazyme, Nanjing, China) were used for real-time PCR in a Prism 7500 real-time thermocycler (Applied Biosystems, Foster City, CA, USA) according to the manufacturer’s instructions. The results were analyzed by the relative quantitation 2^-ΔΔCT,^ and GAPDH was used as an internal control. The primers (Table S1) for GPX4 and DHODH were designed by Tsingke (Beijing, China). Each group was analyzed in triplicate.

**2.4 Cell transfection**

siRNA molecules (Gene Pharma, China) were used to knockdown GPX4 or DHODH expression (Table S2). siRNAs were transfected into cells using Lipofectamine 3000 (Invitrogen, USA) following the manufacturer’s instructions. The sequence of multi-siRNAs was listed in Table S3.

**2.5 Immunofluorescence staining**

Cells for immunofluorescence were seeded on glass plates at 37 °C overnight. The cells were then washed twice with cold PBS, fixed in 4% paraformaldehyde for 20 min, permeabilized with 0.2% Triton X-100 in PBS for 30 min at room temperature, and blocked with goat serum at room temperature for 20 min. After blocking, cell samples were incubated with primary antibody overnight at 4 °C. Then, the nuclei were counterstained with 4’-6-diamidino-2-phenylindole (DAPI, Invitrogen, USA). Images were captured using a confocal microscope (Olympus, Japan).

**2.6 Kaplan–Meier plotter analysis**Survival analysis in HCC patients who received sorafenib treatment was performed on the Kaplan–Meier plotter website (www.kmplot.com), an online database that can assess the effect of 54,675 genes on the prognosis of breast cancer, liver cancer, lung cancer and gastric cancer patients.

**2.7 GEO Datasets**

GSE109211 is published array data of human hepatocellular carcinoma. Tumor tissues from 64 patients (sorafenib responder, n=21; sorafenib nonresponder, n=43) who received sorafenib treatment were collected. Ferroptosis suppressor genes (GPX4, DHODH, FTH, FTL, NFS1, NQO1) were used in the GSVA analysis.

**2.8 Co-culture assay**

The co-culture assay was established using transwell membranes (pores 0.4 μm, Merck Millipore, USA) in a 24-well format. 293T cells transfected with corresponding plasmid and siRNAs were on the up-chamber and then HepG-2 cells below the membranes were ready for further cytological experiments after co-culture.

**3. Supplementary tables**

**Table S1: The sequence of primer sets for qPCR**

| ID | Forward | Reverse |
| --- | --- | --- |
| GPX4 | GAGGCAAGACCGAAGTAAACTAC | CCGAACTGGTTACACGGGAA |
| DHODH | GTTCTGGGCCATAAATTCCGA | TCTGGGTCTAGGGTTTCCTTC |

**Table S2: The sequence of siRNAs**

| **ID** | **sense（5'-3'）** | **antisense（5'-3'）** |
| --- | --- | --- |
| GPX4#1 | GGAGUAACGAAGAGAUCAA | UUGAUCUCUUCGUUACUCC |
| GPX4#2 | GCCAUCAAGUGGAACUUCA | UGAAGUUCCACUUGAUGGC |
| GPX4#3 | GACCGAAGUAAACUACACU | AGUGUAGUUUACUUCGGUC |
| DHODH#1 | GGGCCAUAAAUUCCGAAAU | AUUUCGGAAUUUAUGGCCC |
| DHODH#2 | CGGGAUUUAUCAACUCAAA | UUUGAGUUGAUAAAUCCCG |
| DHODH#3 | CAGGUAUGGAUUUAACAGU | ACUGUUAAAUCCAUACCUG |
| Scramble | UUCUCCGAACGUGUCACGU | ACGUGACACGUUCGGAGAA |

**Table S3: The sequence of multi-siRNAs**

| **ID** | **sense（5'-3'）** | **antisense（5'-3'）** |
| --- | --- | --- |
| multi-siRNA#1 | GGAGUAACGAAGAGAUCAACAAUGGGCCAUAAAUUCCGAAAU | AUUUCGGAAUUUAUGGCCCAUUGCACUUGAUCUCUUCGUUACUCC |
| multi-siRNA#2 | GGAGUAACGAAGAGAUCAACAAGGGCCAUAAAUUCCGAAAU | AUUUCGGAAUUUAUGGCCCUUGAUCUCUUCGUUACUCC |
